# Emerging Roles of De Novo Proline Biosynthesis in Human Diseases

**DOI:** 10.1096/fba.2025-00147

**Published:** 2025-11-05

**Authors:** Ethan Pei, Junfeng Ma

**Affiliations:** ^1^ Department of Oncology, Georgetown Lombardi Comprehensive Cancer Center Georgetown University Medical Center Washington DC USA; ^2^ Richard Montgomery High School at Rockville of Maryland Rockville Maryland USA

**Keywords:** biochemistry, de novo synthesis, metabolism, proline

## Abstract

De novo proline synthesis is a highly conserved and essential biochemical pathway in mammals. Beyond serving as a fundamental building block for proteins, proline also plays key roles in diverse cellular functions and maintaining tissue homeostasis. Over the past decade, accumulating evidence has underscored the significance of this pathway in regulating critical cellular processes, including redox balance, cell growth, signal transduction, and the synthesis of nucleotides and proteins, as well as overall cellular metabolism. The biosynthesis of proline is tightly controlled by multiple evolutionarily conserved mechanisms to ensure proper cellular function. Importantly, disruptions in proline metabolism—particularly changes in the activity or expression of enzymes involved in its synthesis and degradation—have been implicated in the onset and progression of several diseases, notably cancer and fibrosis. In this review, we highlight recent advances in understanding the regulation of de novo proline synthesis. We also examine how dysregulation of this pathway contributes to disease development and influences therapeutic outcomes. Finally, we explore the therapeutic potential of targeting proline metabolism in disease treatment.

## Graphical abstract

De novo proline synthesis, a multi‐step biochemical pathway starting from glutamate, supplies essential protein building blocks. It supports redox balance, cell proliferation, signal transduction, and nucleotide and protein biosynthesis. This tightly regulated pathway, governed by conserved mechanisms, is disrupted in diseases such as cancer and fibrosis, mainly through enzyme dysregulation. Targeting key enzymes in proline metabolism offers significant therapeutic potential.

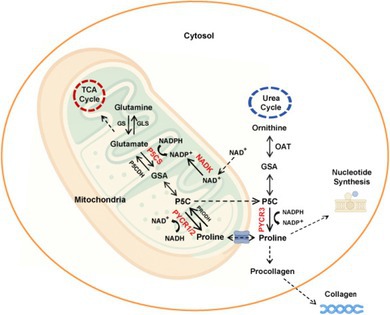

## 1 Introduction

**FIGURE 1:**
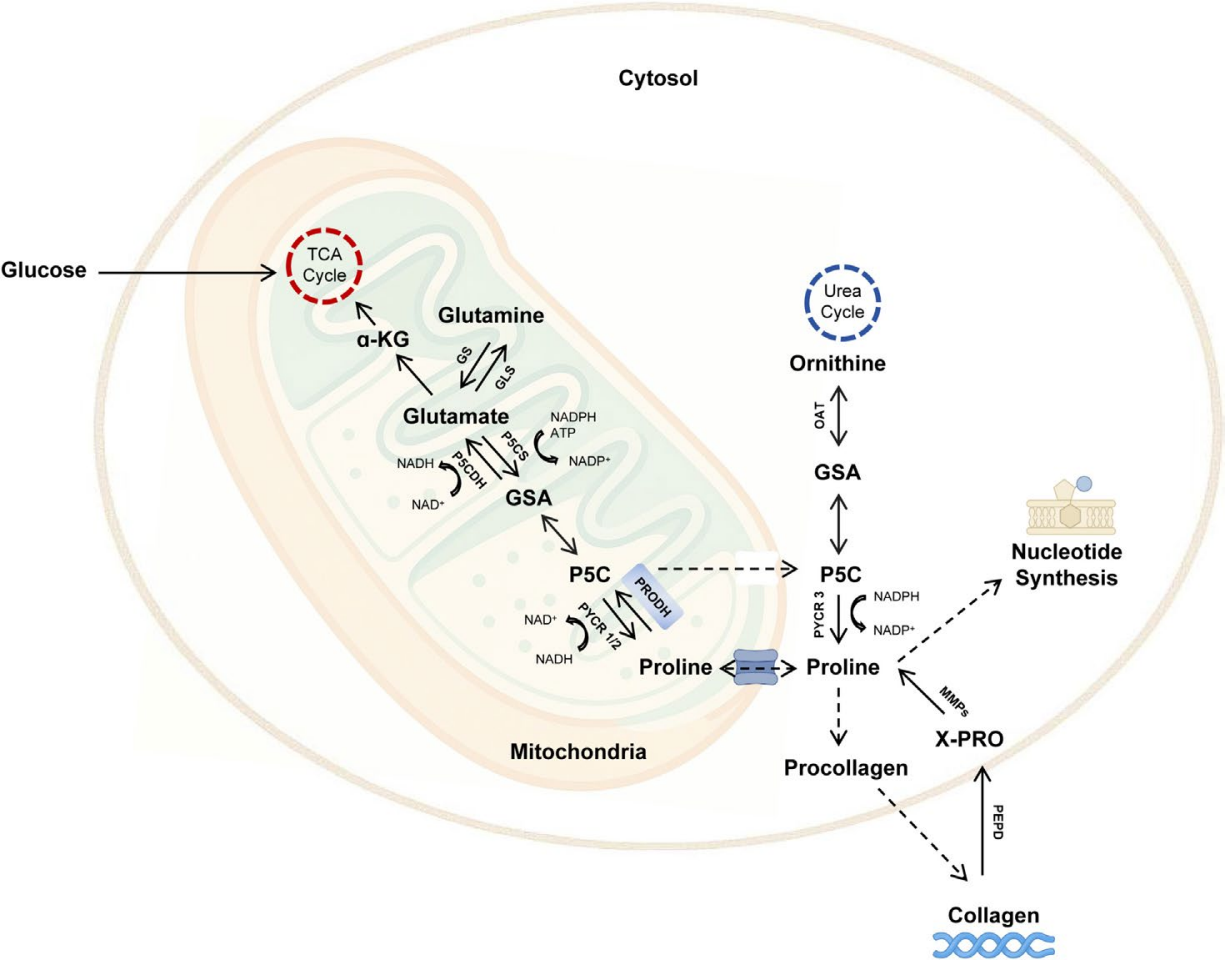
De novo proline biosynthetic pathway. Proline is essential for collagen and nucleotide synthesis, and its biosynthesis involves the reduction of pyrroline‐5‐carboxylate (P5C), a precursor that can be derived via two distinct pathways: From glutamine in the mitochondria and from ornithine in the cytosol. Glutamate and ornithine are converted to glutamate‐γ‐semialdehyde (GSA) by pyrroline‐5‐carboxylate synthetase (P5CS) and ornithine aminotransferase (OAT), respectively. GSA spontaneously cyclizes to form P5C, which is then reduced to proline by pyrroline‐5‐carboxylate reductase isoforms 1, 2, or 3/L (PYCR1, PYCR2, or PYCR3/L). Proline can also be recycled through the action of proline dehydrogenase 1 (PRODH), contributing to metabolic flexibility.

Proline occupies a distinct position in the amino acid pool during protein biosynthesis, largely due to the unique structure of its backbone [1]. Unlike other amino acids, proline's α‐amino group is incorporated into a pyrrolidine ring, making it the only proteinogenic secondary (imino) amino acid [2]. This structural peculiarity underpins its specialized metabolic pathways. Endogenous synthesis of proline mainly proceeds through two routes: the glutamate pathway and the ornithine pathway [3] (Figure 1). The glutamate‐derived route predominates in most tissues and involves a stepwise conversion of glutamate into proline. In contrast, the ornithine pathway becomes particularly relevant under conditions of elevated extracellular proline, indicating a potential salvage or compensatory function [4]. These two biosynthetic routes—along with salvage mechanisms—converge at a common intermediate, pyrroline‐5‐carboxylate (P5C), a central metabolite in proline metabolism [3, 5]. Importantly, de novo proline synthesis is critical not only for the production of structural proteins like collagen, but also for sustaining various cellular functions and preserving tissue homeostasis [6] (Figure 1).

Over the past decade—especially in the last 5 years—significant progress has been made in understanding the regulatory mechanisms of proline biosynthesis, including the pivotal role of mitochondrial NADK2 in de novo synthesis, as well as the physiological and pathological functions of proline metabolism [7, 8, 9]. Increasing evidence has linked proline metabolism to cancer and various human diseases. Numerous studies have demonstrated a cell‐autonomous role for both proline synthesis and degradation in promoting cancer cell growth and survival [10]. However, within the in vivo context, cancer cells reside in a complex tumor microenvironment (TME) composed of a collagen‐rich extracellular matrix (ECM) and diverse stromal components, including cancer‐associated fibroblasts (CAFs), endothelial cells, and immune cells [11]. Proline metabolism not only supports collagen production but also modulates interactions between cancer cells and the TME, thereby influencing tumor progression and therapeutic outcomes [12]. In this review, we highlight recent advances in the regulation of de novo proline synthesis, explore how its dysregulation contributes to disease development and treatment resistance—particularly in cancer—and evaluate the therapeutic potential of targeting this metabolic pathway.

## 2 De Novo Proline Biosynthesis in Cells

Proline de novo synthesis is an evolutionarily conserved and energy‐intensive process that relies on glutamate or ornithine as key metabolic precursors [13] (Figure 1). Glutamine plays a central role by supplying carbon and nitrogen to the TCA cycle and supporting the synthesis of essential biomolecules, including proline [14, 15]. The glutamate–glutamine cycle also contributes to nitrogen homeostasis by detoxifying excess ammonia [16].

The biosynthetic pathway begins with the formation of glutamate‐γ‐semialdehyde (GSAL), catalyzed by pyrroline‐5‐carboxylate synthase (P5CS) in the mitochondria or ornithine aminotransferase (OAT) in the cytosol (Figure 1). GSAL spontaneously cyclizes into pyrroline‐5‐carboxylate (P5C), which is then reduced to proline by pyrroline‐5‐carboxylate reductase (PYCR) [17]. In humans, three PYCR isozymes—PYCR1, PYCR2, and PYCR3 (also known as PYCRL)—catalyze this step. PYCR1 and PYCR2 are mitochondrial and functionally redundant, whereas PYCR3 is localized in the cytosol [18]. Although P5CS is NADPH‐dependent, PYCR1 and PYCR2 exhibit a preference for NADH over NADPH, suggesting distinct regulatory mechanisms across subcellular compartments [19] (Figure 1).

Proline residues within proteins can also undergo post‐translational modifications; for example, it is hydroxylated by prolyl‐4‐hydroxylases (P4Hs) during collagen biosynthesis [20]. Additionally, proline metabolism contributes to the synthesis of important biomolecules, including ribonucleotides and pyridine nucleotides, which are essential for nucleic acid production and cellular energy transfer [21]. In catabolic pathways, proline is oxidized back to P5C by proline dehydrogenase (PRODH/POX). P5C is subsequently converted either to glutamate by pyrroline‐5‐carboxylate dehydrogenase (P5CDH) or to ornithine by OAT, linking anabolic and catabolic branches of proline metabolism [4]. In some cases, proline undergoes hydroxylation by prolyl‐4‐hydroxylases (P4Hs) and contributes to collagen biosynthesis [20].

## 3 The Regulatory Mechanisms of Proline De Novo Synthesis

To sustain cellular homeostasis during proliferation, cells must continuously replenish intracellular proline pools in proportion to their growth and division rates. Accordingly, de novo proline biosynthesis is subject to tight and multifaceted regulation. Recent studies have elucidated several layers of control that coordinate proline production with metabolic and proliferative cues. In this section, we highlight four major regulatory mechanisms governing proline biosynthesis: (1) modulation of mitochondrial NADP(H) availability, which affects the redox balance and enzymatic activity; (2) transcriptional regulation of genes encoding key biosynthetic enzymes; (3) post‐translational modifications of rate‐limiting enzymes; and (4) protein–protein interactions that influence enzyme stability, localization, and activity [8, 9, 22, 23, 24].

### 3.1 Regulation of Mitochondrial NADP(H)

**FIGURE 2:**
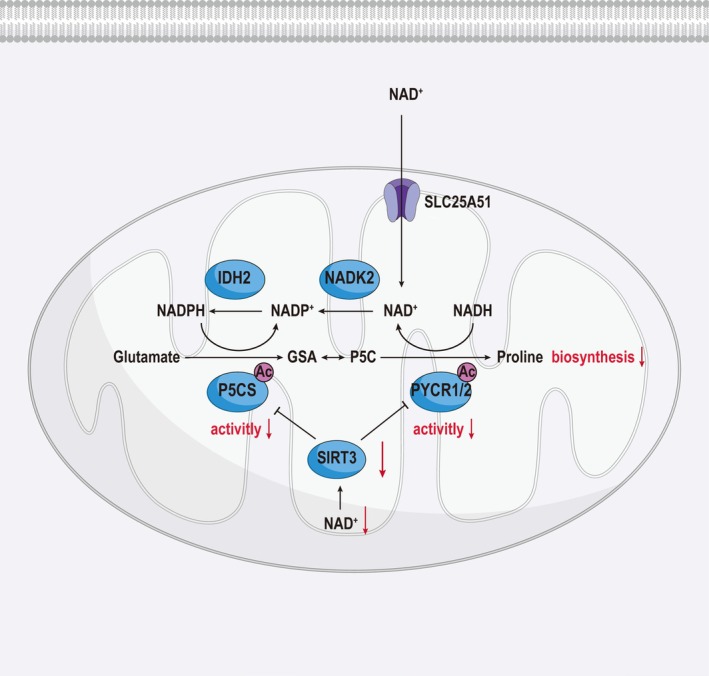
Regulation of mitochondrial NADP(H) in de novo proline biosynthetic pathway. Proline biosynthesis is closely regulated by mitochondrial redox balance and cofactor availability. NADK2 catalyzes the phosphorylation of NAD(H) to generate NADP(H), and its inhibition leads to NADP(H) depletion, which can result in proline auxotrophy. Additionally, SIRT3 negatively regulates proline biosynthesis by deacetylating and reducing the enzymatic activities of P5CS and PYCR1, two key enzymes in the pathway. The mitochondrial NAD^+^ transporter SLC25A51 further contributes to this regulation by mediating the import of NAD^+^ from the cytosol into the mitochondrial matrix. This transport is essential for maintaining mitochondrial redox homeostasis and supporting NAD^+^‐dependent metabolic processes, including those involved in proline synthesis and the electron transport chain.

Nicotinamide adenine dinucleotide phosphate (NADP^+^) and its reduced form, NADPH, are essential redox molecules involved in cellular metabolism. NADP^+^ serves as the oxidized form, whereas NADPH provides the reducing power necessary for macromolecule biosynthesis and defense against oxidative stress. NADPH is compartmentalized into distinct pools in the cytosol and mitochondria [25]. In the cytosol, NADP(H) is generated through the phosphorylation of NAD(H) by NAD kinase 1 (NADK1), whereas in mitochondria, NADP(H) is produced by NAD kinase 2 (NADK2) [26, 27] (Figure 2). Depletion of NADK2 results in a significant reduction of mitochondrial NADP(H) levels, leading to a proline auxotrophy in mammalian cells [8]. Despite no major changes in mitochondrial folate metabolism, TCA cycle activity, or oxidative stress, NADK2‐deficient cells show impaired proliferation in minimal medium, a defect that can be rescued by proline supplementation [7, 28].

On the mechanistic level, proline biosynthesis occurs in the mitochondria, where glutamine‐derived glutamate is converted into pyrroline‐5‐carboxylate (P5C) by pyrroline‐5‐carboxylate synthase (P5CS) [29]. P5C is then further reduced to proline by mitochondrial pyrroline‐5‐carboxylate reductases (PYCR1 and PYCR2) [30]. Although P5CS is NADPH‐dependent, PYCR1 and PYCR2 exhibit a higher affinity for NADH than for NADPH. The loss of NADK2 and the consequent depletion of mitochondrial NADP(H) block P5CS activity, thus impairing the reduction of glutamate to P5C and hindering proline biosynthesis [31] (Figure 2).

Previous studies also have demonstrated that SLC25A51, a mitochondrial NAD^+^ transporter, plays a critical role in the regulation of proline biosynthesis through its mediation of mitochondrial NAD^+^ translocation [32, 33] (Figure 2). SLC25A51 facilitates the import of NAD^+^ from the cytosol into the mitochondrial matrix, thereby maintaining mitochondrial redox homeostasis and supporting essential metabolic processes, including the electron transport chain and NAD^+^‐dependent enzymatic reactions [34] (Figure 2). This NAD^+^ transport is especially crucial for proline biosynthesis. Within the mitochondria, NAD^+^ is required for the production of NADPH, a key cofactor for the enzymatic conversion of glutamate into pyrroline‐5‐carboxylate (P5C) via pyrroline‐5‐carboxylate synthase (P5CS) [35]. Thus, by regulating mitochondrial NAD^+^ availability, SLC25A51 indirectly controls the NADPH‐dependent steps of proline synthesis.

### 3.2 Regulation of the Key Enzymes at Transcriptional Level

P5CS is a key enzyme in the proline biosynthesis pathway and is encoded in humans by the ALDH18A1 gene, a member of the aldehyde dehydrogenase family located on Chromosome 10. The gene spans approximately 50 kb and contains 18 exons [35]. ALDH18A1 transcription is tightly regulated. Its promoter region includes predicted binding sites for the tumor suppressor p53 and the glucocorticoid response element (GRE), along with an additional p53 binding site located within the first intron. Studies have shown that P5CS expression is influenced by both growth hormones and p53 [17]. In addition, age‐dependent downregulation of ALDH18A1 in mouse brain tissue has been associated with progressive hearing loss [36]. Recent findings further suggest that the transcription factor myeloid zinc finger 1 (MZF1) can upregulate ALDH18A1 expression, and that elevated proline levels may contribute to the aggressive phenotype of neuroblastoma (NB) cells [37].

**FIGURE 3:**
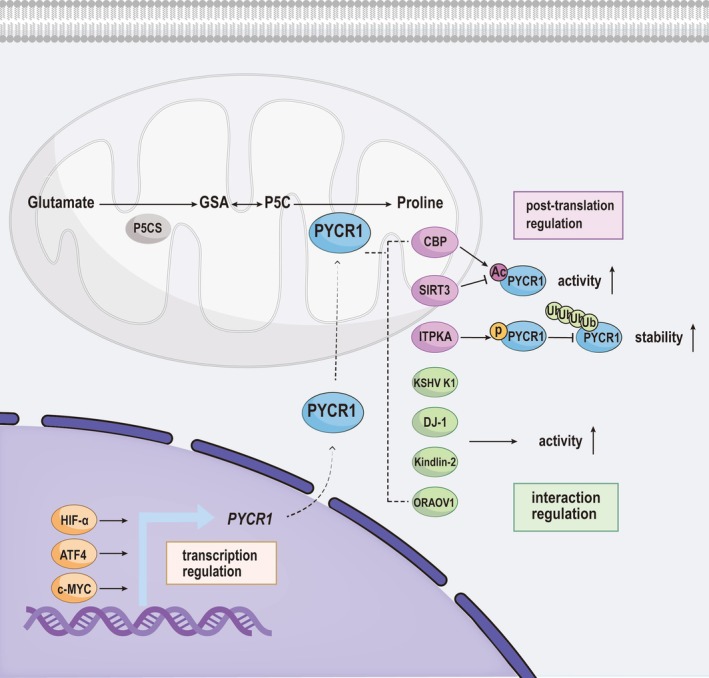
Transcriptional and post‐translational regulation of the key enzymes in the proline biosynthetic pathway. PYCR1, a key enzyme in proline biosynthesis, is regulated at multiple levels, including transcription, post‐translational modifications, and protein–protein interactions. Transcription factors such as HIF‐α, ATF4, and c‐MYC directly upregulate PYCR1 gene expression. At the post‐translational level, PYCR1 activity is enhanced by acetylation through CBP and inactivated by deacetylation via SIRT3. Moreover, ITPKA‐mediated phosphorylation of PYCR1 inhibits its ubiquitination and degradation, thereby stabilizing the protein. PYCR1 function is also modulated through interactions with various proteins—including KSHV K1, DJ‐1, kindlin‐2, and ORAOV1—all of which contribute to the enhancement of PYCR1‐mediated proline synthesis.

PYCR1, another key enzyme in the proline biosynthesis pathway, is regulated by multiple transcription factors involved in cellular stress responses, metabolic adaptation, and oncogenesis (Figure 3). c‐Myc, a well‐characterized oncogenic transcription factor, directly upregulates PYCR1 expression, thereby linking proline metabolism to tumor growth [38]. In parallel, ATF4, a central effector of the integrated stress response, induces PYCR1 under conditions of oxidative or amino acid stress [39] (Figure 3). Additionally, HIF‐1α, the master regulator of hypoxic signaling, enhances PYCR1 transcription in low‐oxygen environments, promoting proline production to sustain redox homeostasis and cell survival [40] (Figure 3). Collectively, these findings underscore how PYCR1 is embedded within broader transcriptional networks that enable cells to adapt to environmental stressors and oncogenic signals.

### 3.3 Regulation of the Key Enzymes at Post‐Translational Level

Recent studies have demonstrated that the activity of PYCR1, a key enzyme in proline biosynthesis, is tightly regulated through post‐translational modifications (PTMs), which significantly impact its stability, enzymatic activity, and oncogenic potential [41] (Figure 3). One such regulator is inositol‐trisphosphate 3‐kinase A (ITPKA), which is aberrantly overexpressed in glioma and correlates with poor patient prognosis. Functional studies have demonstrated that ITPKA promotes glioma cell proliferation and invasion, underscoring its role in tumor progression. Mechanistically, ITPKA directly binds to PYCR1 and phosphorylates it at serine 29. This post‐translational modification inhibits Stub1‐mediated ubiquitination, thereby preventing proteasomal degradation and enhancing PYCR1 protein stability. The stabilization of PYCR1 through serine 29 phosphorylation represents a key mechanism by which ITPKA sustains elevated proline biosynthesis and contributes to the oncogenic phenotype of glioblastoma [42] (Figure 3).

In addition to phosphorylation, PYCR1 is also regulated by acetylation. CBP (CREB‐binding protein) acetylates PYCR1, leading to suppression of tumor cell growth. In contrast, SIRT3, a mitochondrial deacetylase, removes this modification, thereby increasing PYCR1 enzymatic activity. Enhanced proline production through SIRT3‐mediated deacetylation has been shown to promote tumor cell proliferation [43] (Figure 3).

### 3.4 Protein–Protein Interactions Regulating PYCR1 Function

In addition to post‐translational modifications, protein–protein interactions play a critical role in regulating PYCR1 function (Figure 3). Mechanical changes in the extracellular matrix (ECM), such as matrix stiffening, promote the translocation of kindlin‐2 from the cytosol to the mitochondria, where it directly binds to PYCR1. This interaction stabilizes PYCR1, enhances proline synthesis, and promotes the proliferation of lung adenocarcinoma cells. In contrast, kindlin‐2 knockdown reduces PYCR1 levels, leading to suppressed tumor growth and improved survival outcomes [11].

Several other proteins also interact with PYCR1 to enhance its proline‐synthetic activity. These include DJ‐1, ORAOV1 (oral cancer overexpressed 1), and KSHV K1 (Kaposi's sarcoma‐associated herpesvirus protein), which collectively contribute to antioxidant defense and tumor development [44, 45, 46] (Figure 3). Additionally, PINCH‐1 has been shown to strengthen the interaction between kindlin‐2 and PYCR1, further promoting proline production and tumor progression [24] (Figure 3).

## 4 Dysregulation of De Novo Proline Biosynthesis in Cancer and Other Diseases

The de novo biosynthesis of proline is a metabolically conserved pathway essential for redox homeostasis, collagen production, and mitochondrial function. This pathway primarily involves three enzymatic steps mediated by ALDH18A1 (which encodes P5CS) and members of the PYCR enzyme family (PYCR1, PYCR2, and PYCR3/PYCRL). Increasing evidence implicates dysregulation of this pathway in a range of pathological conditions, including cancer, fibrotic diseases, metabolic syndromes, and neurological disorders. Here, we summarize key findings linking altered proline metabolism to human disease, highlighting mechanistic insights and therapeutic implications [21, 47].

### 4.1 Cancer

Alterations in proline biosynthesis have been widely observed in cancer, where they support tumor growth, redox balance, and survival under metabolic stress. In hypoxic tumor microenvironments, ALDH18A1 expression is upregulated while PRODH2 activity is reduced, leading to hydroxyproline accumulation [48]. Hydroxyproline inhibits prolyl hydroxylases, stabilizing HIF‐1α and promoting cancer cell survival, notably in hepatocellular carcinoma (HCC) [21, 47].

Among proline biosynthetic enzymes, PYCR1 has emerged as a central regulator of cancer metabolism. It is frequently overexpressed in diverse malignancies—including breast cancer, non‐small cell lung cancer (NSCLC), and prostate cancer—where it promotes proliferation, inhibits apoptosis, and facilitates invasion and epithelial–mesenchymal transition (EMT). Mechanistically, PYCR1 influences oncogenic pathways, such as ERK, p38 MAPK, and cyclin D1/Bcl‐2 signaling. Genetic or pharmacologic inhibition of PYCR1 results in reduced tumor growth and increased cell death in preclinical models, underscoring its potential as a therapeutic target [11, 49, 50].

Furthermore, mitochondrial NADP(H) is essential for proline biosynthesis, and NAD kinase 2 (NADK2) plays a key role in maintaining this redox pool. NADK2 converts NAD(H) to NADP(H), supplying reducing equivalents required for PYCR‐mediated proline production. Inhibition of NADK2 depletes NADP(H), induces proline auxotrophy, and impairs tumor cell proliferation—highlighting a novel metabolic vulnerability in rapidly dividing cells [8].

### 4.2 Fibrotic Diseases

Proline is a key substrate for collagen synthesis, and its dysregulated production contributes to pathological fibrosis. In idiopathic pulmonary fibrosis (IPF), liver fibrosis, and systemic sclerosis, proline biosynthesis is upregulated to meet the excessive collagen demand of activated fibroblasts. Elevated expression of ALDH18A1, PYCR1, and NADK2 has been reported in fibrotic tissues and is associated with disease severity [9, 10]. For example, increased NADK2 expression in IPF lungs correlates with reduced pulmonary function and enhanced fibrogenic activity, suggesting a proline‐driven mechanism of extracellular matrix (ECM) accumulation and tissue stiffening. Targeting proline metabolism in fibrotic diseases is a promising strategy to modulate ECM dynamics and attenuate fibrotic progression [51, 52].

### 4.3 Metabolic and Mitochondrial Disorders

Loss‐of‐function mutations in proline biosynthetic enzymes result in rare but severe inherited metabolic and mitochondrial disorders. PYCR1 mutations cause autosomal recessive cutis laxa type IIB (ARCL2B), characterized by connective tissue defects, growth retardation, and craniofacial abnormalities [53]. Mutations in PYCR2 are linked to HIDEA syndrome, a neurodevelopmental disorder featuring hypotonia, intellectual disability, epilepsy, and structural brain abnormalities [54]. These conditions illustrate the essential role of proline metabolism in maintaining mitochondrial integrity, energy homeostasis, and structural protein biosynthesis. Notably, mitochondrial dysfunction in these disorders reflects the reliance of oxidative metabolism on intact proline biosynthetic capacity [53, 54].

### 4.4 Neurological Implications

Emerging data suggest that proline metabolism also plays a crucial role in brain development and neuronal function. Proline serves not only as a structural component but also as a precursor for neurotransmitters and an antioxidant modulator in neurons. PYCR2 deficiency, in particular, leads to profound neurodevelopmental phenotypes, including microcephaly, brain atrophy, and severe intellectual disability [54]. Although the direct mechanistic links between proline biosynthesis and common neurodegenerative diseases remain underexplored, mitochondrial dysfunction caused by impaired proline metabolism may contribute to broader neuropathological states. Future research should investigate whether modulating this pathway could offer neuroprotective benefits [54].

## 5 Perspectives and Conclusions

The de novo proline biosynthesis pathway is increasingly recognized as a pivotal metabolic hub involved in cancer, fibrosis, metabolic disorders, and neurological diseases. Key enzymes such as PYCR1, ALDH18A1, and NADK2 have emerged as promising therapeutic targets. Preclinical studies indicate that inhibiting PYCR1 can suppress tumor growth, while targeting NADK2 may disrupt mitochondrial NADP(H) pools essential for proline production in proliferative cells. Similarly, modulating ALDH18A1 activity could attenuate both tumor progression and fibrotic remodeling. However, proline metabolism is fundamental to normal cellular functions, posing challenges to therapeutic targeting in terms of selectivity and safety. The metabolic heterogeneity among tumors and the adaptability of cancer cells necessitate precise strategies, including the development of selective inhibitors and combination therapies tailored to specific disease contexts.

Despite significant advances in characterizing the enzymatic machinery of proline biosynthesis, the regulatory mechanisms controlling this pathway in various physiological and pathological states remain incompletely understood. This gap is largely due to limitations in tracing proline synthesis flux and post‐translational modifications within different cell types and disease models. Future studies employing advanced methodologies—such as stable isotope tracing, metabolomics, proteomics, and the use of animal and clinical models—are essential to unravel the complex regulation of proline metabolism.

Although clinical translation of proline metabolism‐targeted therapies is still in its infancy, a comprehensive understanding of its role within broader metabolic networks will be critical. Such knowledge will help anticipate potential side effects and identify patient populations most likely to benefit. Given the metabolic diversity across tumor types, systematic investigation into proline metabolic dysregulation promises to deliver new diagnostic biomarkers and therapeutic strategies for cancer and other proline‐related diseases.

In summary, while de novo proline biosynthesis represents a promising therapeutic avenue, realizing its full clinical potential will require continued interdisciplinary research focused on mechanistic understanding, biomarker discovery, and precision‐targeted interventions.

## Author Contributions

E. Pei wrote the original draft and drew the figures. J. Ma revised the manuscript and figures. All authors reviewed the manuscript and approved the submitted version.

## Ethics Statement

The authors have nothing to report.

## Consent

The authors have nothing to report.

## Conflicts of Interest

The authors declare no conflicts of interest.

## Data Availability

The authors have nothing to report.
